# Can Personalization Persuade? Study of Notification Adaptation in Mobile Behavior Change Intervention Application

**DOI:** 10.3390/bs12050116

**Published:** 2022-04-19

**Authors:** Amadej Jankovič, Tine Kolenik, Veljko Pejović

**Affiliations:** 1 Faculty of Computer and Information Science, University of Ljubljana, 1000 Ljubljana, Slovenia; amadej.jankovic@gmail.com (A.J.); veljko.pejovic@fri.uni-lj.si (V.P.); 2 Department for Intelligent Systems, Jožef Stefan Institute, 1000 Ljubljana, Slovenia; 3 Jožef Stefan International Postgraduate School, 1000 Ljubljana, Slovenia

**Keywords:** digital behavior change intervention, motivation, personality, persuasive technology, user study

## Abstract

The growing ubiquity of smartphones and the ease of creating and distributing applications render the mobile platform an attractive means for facilitating positive behavior change at scale. Within the smartphone as a behavior change support system, mobile notifications play a critical role as they enable timely and relevant information distribution. In this paper we describe our preliminary investigation of the persuasiveness of mobile notifications delivered within a real-world behavior change intervention mobile app, which enabled users to set goals and define tasks related to those goals. The application aimed to motivate the users with notifications belonging to one of two groups—tailored and non-tailored, seeing them as sparks in the Fogg Behavior Model and personalizing them according to the users’ Big Five personality traits. Results indicate that customized messages may work for some individuals while working poorly for others. When analyzing users as a single group, no significant differences were observed, but when proceeding with the analysis on the individual level we found seven users whose personality traits notifications interact with in interesting ways. Our results offer two general insights: (1) Using personality-tailored messaging in a dynamic mobile domain as opposed to a static domain leads to different outcomes, and it seems that there is no one-to-one mapping between domains; (2) A major reason for most of our hypotheses being false may be that messages that are deemed as persuasive on their own are not what persuades people to perform an action. Unlike the clear-cut findings observed in other domains, we discover a rather nuanced relationship between the personalization and persuasiveness that calls for further exploration at the individual participant level.

## 1. Introduction

Mobile phones represent the most direct ubiquitous information delivery platform in the world. The pervasiveness, encapsulated by more than three billion active users, who always carry their phones and display a tight user-device bond, has been recently recognized and harnessed by digital behavior change intervention (dBCI) applications [1]. DBCIs belong to the wider area of persuasive technology (PT) [2] and behavior change support systems [3]. These applications aim to facilitate positive behavior change in their users, and to date have targeted domains as diverse as weight loss [4], stress and depression [5,6], substance abuse [7], and even green behavior [8]. Besides the ubiquitous emotional distress in populations affected by the Covid-19 pandemic [9], depression, obesity, and type-2 diabetes that have ravaged humankind, affecting more than 1.3 billion people worldwide. To address these issues in effective ways, the development of digital tools allowing mass behavior-based treatment of preventable diseases is of key importance. The increasing cost of face-to-face behavioral therapies, the lack of qualified health workers, and diminishing national healthcare budgets further exemplify the need for a low-cost ubiquitous dBCI delivery method [10].

Push notifications on mobile phones are a particularly powerful instrument allowing a sender to initiate interaction with a recipient in an asynchronous manner. Notifications allow timely behavior guidance dissemination, nudging, and experience sampling, which could make them of utmost importance in the mobile dBCI realm. Nevertheless, little is known about the effectiveness of such remote asynchronous messages. Past research, outside the scope of dBCI, demonstrates that the perception of mobile notifications as well as a user’s reaction to a notification depend on numerous factors, including the message timing, the content of the message, and the relationship between the sender and the receiver, to name a few [11]. In the field of dBCI, outside of a few studies on the role of the notification timing and content [12,13], mobile notifications remain rather underexplored. The usage of push notifications as a viable vector for delivering dBCI information, particularly in the form of framing the persuasive dBCI content to individual personalities in a successful manner, is yet to be explored.

This paper presents the initial study of the efficiency of personality-adapted mobile notifications for behavior change elicitation in a mobile dBCI app, basing our tailoring on the Big Five personality traits model [14]. Refraining from a narrow use case we designed a general life-coaching mobile app, distributed it to 27 participants, and over two weeks monitored users’ reactions to mobile notifications, users’ attitudes towards the notifications, as measured via the experience sampling method, and compliance with the pre-set behavior change goals. In contrast to the findings from the non-mobile advertising domain [15], we find a much more nuanced relationship between the notification content and the user behavior. It points to a possibility that transferring findings on the influence of tailored messaging from one setting to the other (domain-transferability) does not translate as expected. What is more, our research points to a possibility that perceived persuasiveness of messages does not correspond to people being persuaded to perform an action. While we discover that certain personality traits, such as neuroticism, indicate a likely preference for personality-tailored content, the link between the content and the reaction to a message is, in general, not as clear and requires further multifaceted examination. Apart from our findings, contribution also encompasses a novel dataset, which is made publicly available for further inspection and cross-examination.

## 2. Related Work

The link between personality and persuasiveness may be examined inside or outside the domain of technology. In the marketing domain, Hirsh et al. [15] conclude that considering only the dominant personality dimension when tailoring messages for persuasion is a successful strategy. The authors reinforced the notion that there is a uniform influence of dimensions over users when focusing on their dominant one, and provide a straightforward framework for future research on the interplay between tailored persuasion and personality. Kaptein et al. [16] introduced the idea of richer, multi-dimensional, persuasion profiles—a way of constructing the estimates of the expected effects of persuasive methods for specific individuals. The persuasion profile can be generated explicitly, e.g., via a questionnaire prepared in advance, or implicitly by adapting the system based on users’ interaction with it. Through small-scale real-world studies the authors demonstrate that messages tailored to the persuasion profiles indeed positively impact the outcome of persuasive campaigns, including those related to dBCI. Our study is informed by the above findings: we rely on the dominant dimension tailoring paradigm from Hirsh et al., but in a digital and dynamic context of mobile computing, as Kaptein et al. showed that persuasion can work in such contexts as well. Through these two foundations, we want to explore how persuasion, based on the dominant personality dimension of the Big Five personality traits model, translates from a static environment (of, e.g., classic marketing) into a digital dynamic environment of mobile behavior change interventions.

Such a result indicates that our study, too, could detect a link between persuasiveness and personalization. Unlike Kaptein et al., however, we rely on the Big Five personality traits for message tailoring. The ability to quickly infer a user’s personality traits, either via direct tests or proxies, such as online social network-related behavior, significantly increases the practicality of message personalization.

In PT research, specific domain-based focus (e.g., health) is a lot more commonly investigated than persuasion for general tasks that span over a wide range of domains. The Big Five personality traits model is not uncommonly researched, but preferred behavior change theories include Cialdini’s principles of persuasion [17] and the Fogg behavioral model [18]. This is especially true when it comes to tailored messaging, used in our study, which is a common technique that leads to favorable outcomes [19].

A large body of research evaluates how PTs appear to their users [20], without evaluating whether these techniques actually persuade people to perform actions (at most, participants are presented with hypothetical scenarios [21]). Moreover, research encompassing the Big Five personality traits, general use cases, and tailored messaging is scarce, with the exception of Halko et al. [22] study that explored the personality–persuasiveness relationship in the realm of health-promoting mobile applications. The results of this study suggest that conscientious individuals may be more prone to socially-based technologies, extraverts may not desire PTs as they have a tendency to have strong social networks, neurotic individuals prefer working alone to achieve their goals, open individuals are influenced by competitive and authoritative approaches in messaging, and agreeable individuals do not seem to respond well to PTs. However, whether personality-tailored mobile messaging can be efficient for general-purpose persuasiveness remains to be seen.

From the available research it is therefore not certain whether the personality-tailored messaging for general purposes can be efficiently implemented in a dynamic, mobile environment using the dominant dimension paradigm as it works in the static environment. We base our research design on this inquiry.

With profiling of the users and adapting the persuasive system to their needs, the idea to look within every individual to guide our persuasive efforts, and apply them to clusters of users susceptible to them, emerges. We designed our study on existing work regarding personality and PT and focused primarily on personalizing the intervention to the most dominant personality dimension of users to explore and gain a deeper understanding of the method’s sufficiency. We do not aim to change personality traits as some studies in this field have proposed [23]. Our aim is specifically limited to investigating whether and how notifications, working as sparks in the Fogg Behavior model and tailored to the Big Five personality traits profiles of the users through the personalization technique outlined in [15], change the users’ behavior in the context of the application.

## 3. Methods and Materials

Our goal is to explore the role of mobile notifications, tailored to personality traits, on inducing behavior change. Restricting our application to a narrow use case would risk drawing conclusions that are relevant for a particular domain only. Thus, we design a more general Android mobile application that enables users to set goals and define tasks relating to those goals (further described in Section 3.3). The application then aims to motivate the users with messages delivered via push notifications, which are the key intervention element of our study. They are constructed to belong to one of the two groups—tailored and non-tailored messages. According to the best practices in persuasive app design, we also include visualizations indicating the percentage of tasks completed and use gamification, in the form of virtual coins, to further motivate the users. However, the visualization and the gamification parts are not central to our study and do not adapt according to a user’s personality or in any other way.

**Figure 1:**
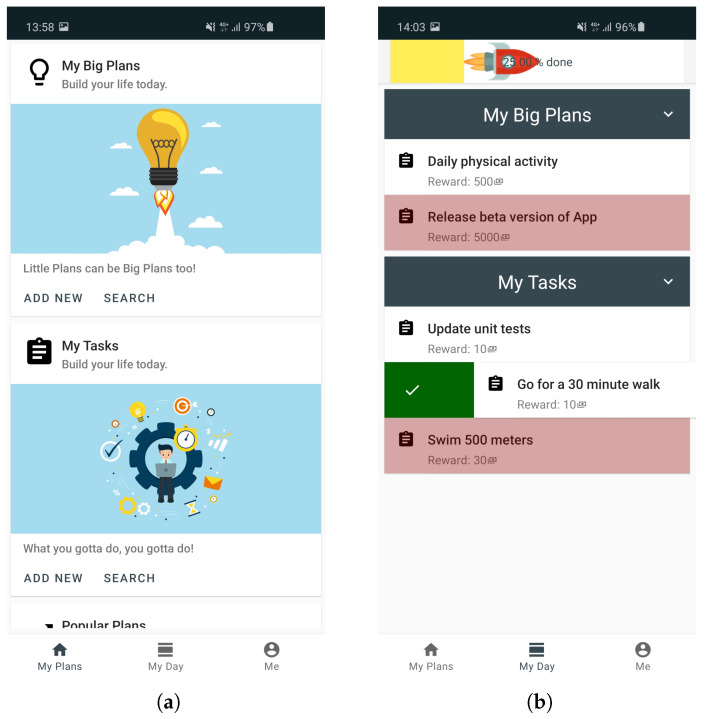
Core screens of our mobile application: (**a**) the landing page; (**b**) overview of specific Big Plans that contain separate tasks; a user assigns a reward to each task, sets a deadline, and can mark tasks as “done”.

The app was implemented for the Android mobile operating system, embraces the Android Material design patterns [24] to ensure “contemporary” look and feel. It relies on mobile sensing to detect activity breakpoints (further elaborated in Section 3.1) in order to deliver messages at the most appropriate times [25]. Sample screens of our applications are displayed in Figure 1. Supplementing the core screens presented on Figure 1, the application used additional 15 activities and fragments, providing users with different functionalities, ranging from the CRUD (create, read, update, delete) operations for their tasks and plans, to modifying their settings related to app usage (eg. setting up do not disturb mode for notifications, setting up their home/work locations, etc.), including the process of account creation, login, authentication, and onboarding with the first time app usage. Results of the BFI-10 personality test were also presented to the user in app.

### 3.1. Motivational Prompts

Within the scope of the Fogg’s Behavior Model [18], push notification messages in our app serve as sparks, i.e., prompts that motivate. We construct them to belong to one of the two groups—*tailored* and *non-tailored* notification messages (TN and NTN respectively). The TN were adapted according to the dominant dimension of a user’s personality. This dimension is determined via an in-app Big Five 10-question personality test (BFI-10) [14] revealing an individual’s personality traits consisting of openness to experience, conscientiousness, extraversion, agreeableness, and neuroticism [26].

We constructed the TN messages in advance and arranged them into five categories—one for each personality dimension. The messages in each category emphasized the most prominent characteristics of that category. The content of the messages in each category was crafted according to the method presented by Hirsh et al. [15] that boosts the persuasive appeal by framing a message according to the recipient’s dominant personality dimension [15]. Thus, rewards and social attention were emphasized for extravert users; communal goals and interpersonal harmony for agreeable users; order and efficiency for conscientious users; uncertainty, danger and threats for neurotic users; and finally, with open users, messages emphasized creativity, innovation and intellectual stimulation. Messages can be accessed at https://gitlab.fri.uni-lj.si/lrk/personality-tailored-notifications/-/blob/master/questions_for_users.pdf (accessed on 20 February 2022).

Finally, previous research has examined the importance of the timing of prompts [27] with the goal of recognizing the “right” moments to send the message. While numerous factors may affect a user’s readiness to react to, and indeed even notice, a notification, a *breakpoint*, such as change in activity, e.g., from sitting to walking or from walking to sitting, is often found to be a favorable moment for interrupting a user [25]. Thus, in our application we harness a built-in Google Activity Recognition classifier to detect activity transitions in a battery-friendly manner and, in case sufficient time has passed since the last notification (1.5 h), a new message is delivered.

### 3.2. Daily Feedback Collection

User experience was measured with the experience sampling method (ESM) [28], one of the most reliable procedures for studying and measuring people’s experiences [28]. In our study ESM was interval-contingent and based on fixed time intervals—each day around 8 PM, users were prompted to complete an in-app questionnaire. The questionnaire included the motivational messages delivered on that day, together with the accompanying task names. The users then had to rate their perceived motivation induced by each of the messages by answering: “The message motivated me for pursuing my goals” using a Likert scale (1—completely disagree, to 5—completely agree).

### 3.3. Recruitment and Data Gathering Campaign

We recruited 27 volunteers (15 male, 11 female, 1 other, age group 20–29 years old) through our personal contacts, which allowed us to have an in-person conversation with each individual. The users were made aware of the data collected by the app, and were given instructions on how to register and use the app. Afterwards, each user completed an in-app personality test from which the percentiles of personality dimensions of a particular user were found. Means and standard deviations used for the percentile computation were taken from a larger study that included a representative sample from a culturally similar population [29]. The personality dimension for which the user was in the highest percentile was then determined as the most dominant and used for TN. Big five personality dimension distribution by dominant personality trait among users was as follows: five users with dominant dimension openness (three males, two females, one other), two users with dominant dimension conscientiousness (two females), no users with extraversion as the most dominant dimension, 11 users with the most dominant dimension agreeableness (seven males, four females), and eight users with the most dominant dimension neuroticism (five males, three females). The users used the app to set goals and tasks related to those goals. When creating tasks, users could choose the difficulty (easy, medium, or hard). After creation, our app randomly assigned either TN or NTN to the task, so that approximately half of the tasks are accompanied with each of the message groups. The in-app daily questionnaires were sent out every evening as described in Section 3.2.

### 3.4. Research Hypotheses

Our study is exploratory by nature. Nevertheless, we formed the core hypotheses beforehand. The goal of these hypotheses was not only to provide structure to our exploration, but also to pave guidelines for future research in the area of push notification content tailoring for dBCIs. The formed hypotheses are presented in Table 1.

### 3.5. Data Overview

**Figure 2:**
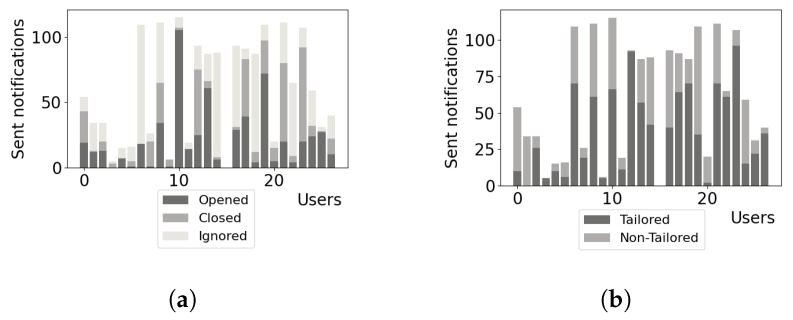
Notification action and tailored/non-tailored notification distribution. (**a**) denotes the distribution of reactions to notifications delivered, (**b**) denotes the proportion of TN delivered to users.

The distribution of reactions to notifications delivered can be seen in Figure 2a with the proportion of TN delivered to users seen in Figure 2b. As observed in the figures, the users did not contribute equally, which was related to a few challenges we faced during data collection—not all users had the same interest in the app usage, some users went on unplanned vacations, and some users had unplanned personal obligations. Some users therefore did not complete their questionnaires for all of the delivered tasks, which was taken into account during the data analysis by constraining the analysis of perceived motivation to tasks with answered questionnaires.

## 4. Results

The hypotheses presented in Section 3.4 target different aspects of persuasiveness and its perception. Here we investigate how the perceived motivation and the task completion differ depending on whether a user had received a TN or an NTN. Finally, we take a closer look at the impact on the most active users in our study. Table 1 summarizes the hypotheses outcomes.

### 4.1. Perceived Motivation of Messages

Previous studies have explored the role of message personalization, but only through storyboard scenarios where users evaluated the persuasiveness potential of a particular message [15]. Our study puts users in actual situations where a persuasive message is received. Thus, by asking the users to assess the motivational power of these messages, we evaluate the actual perceived motivation of persuasive messages and assess our H1.

We code the 5-step Likert scale answers about the message motivational power to a 1 to 5 interval and perform the *t*-test over the per-user average ratings of the two groups of messages in our dataset—TN and NTN. With the application of Bonferroni Correction, the *p*-value produced from tests should fall below 0.004 for statistically significant differences to be detected. The analysis of the whole dataset implies almost no difference (*t*(39) = 0.1, *p* = 0.898) between the ratings of TN and NTN, with TN (*M* = 3.71, *SD* = 1.1) being scored slightly higher than NTN (*M* = 3.66, *SD* = 1.1). However, by significance probability being higher than our threshold, the possibility that there is a difference is not excluded so we proceed with dividing the data according to the users’ demographic and psychographic characteristics, and re-run the analysis. Of all the bisections, the most notable difference was found among the neurotic individuals, with TN (*M* = 3.9, *SD* = 0.7) favored a little over NTN (*M* = 3.5, *SD* = 1.0), *t*(13) = 0.8, *p* = 0.448, which further increased when every individual measurement of scores (instead of a per-person average) for TN (*M* = 4.15, *SD* = 1.12) and NTN (*M* = 3.9, *SD* = 1.3) was considered. The differences indicated subtle preference for TN, however they were not statistically significant, *t*(284) = 1.8, *p* = 0.071.

While our H1 examines the differences in reactions to TN within various groups of users, H6 investigates the differences at the individual user level. We first conduct Levene’s test to infer whether the variances between the ratings of TN and NTN are equal, if so, we proceed with *t*-test otherwise with Welch’s *t*-test. In Table 2 we show the analysis for the seven users for whom the differences between reactions to TN and NTN were found. The symbols in the table are as follows: number ‘1’ represents TN and number ‘2’ represents NTN; the letters C, A, N, O, E represent the personality dimension conscientiousness, agreeableness, neuroticism, openness, and extraversion, respectively; we list the percentile in which the user is for a given dimension, as obtained by the BFI-10 personality test; the ‘*’ symbol denotes that Welch’s *t*-test was used.

This investigation of the seven subjects where the differences between reactions to TN and NTN were found may be important as a meaningful finding may be that using TN or NTN may not be enough to change a behavior. What also needs to be taken into account is a specific personality of a user, and only some personality profiles interact with TN or NTN, while for other personality profiles, there are not differences. While our sample size is small, this indication would provide valid grounds for further research into this direction.

While the differences are not statistically significant with correction applied, the seven users can nevertheless be roughly divided into three groups. The first group contains two males, who have perceived the NTN as more motivating, scoring them higher on average. The second group contains three females, who on average all perceived the TN as well as NTN as motivating. Lastly, the third groups contains one male and one female, who both have highly expressed neuroticism, and have rated the received TN more motivating than NTN. Since we found individuals between whom perceived motivation of messages indicated a subtle difference, we conclude that our H6 is satisfied, and leave further discussion for the penultimate section. As the nature of this study is exploratory, it must be stressed that the conclusions from these results should be taken as a subtle guide to direct further research, rather than a definitive answer.

### 4.2. Impact on Task Completion

A user’s perception of whether a message is persuasive need not correspond to the actual reaction to the message. Indeed, it could be possible that a user actually completes a task despite a nudging message not being welcomed. Thus, in this section we examine the impact of motivational messages on the completion of a task that the message relates to.

With the examination of H2 we find that the proportion of completed tasks was higher in the group of tasks that received TN (*M* = 38.2, *SD* = 33.2) than in the group that received NTN (*M* = 34.6, *SD* = 32.6), however the difference was not significant (*t*(44) = 0.4, *p* = 0.719). The difference remained insignificant (*t*(28) = 0.8, *p* = 0.426) when taking only active individuals who received 40 notifications or more into account. However, bigger difference was observed in active individuals, with completion in the group of tasks that received TN (*M* = 43.6, *SD* = 35.1) being higher than in the group that received NTN (*M* = 33.4, *SD* = 31.5).

In another comparison we analyzed the differences between the completion percentage of tasks for which the user received a notification (regardless of it being TN or NTN) and the completion percentage of tasks for which no notifications were delivered. While the completion is higher for tasks that received the notifications (*M* = 36.1, *SD* = 30.8) than for those that did not receive the notifications (*M* = 27.9, *SD* = 24.8), the difference was not significant (*t*(44) = 1.0, *p* = 0.333), indicating that H3 should be rejected. Again, bigger differences were observed between the group of tasks that received a notification (*M* = 38.4, *SD* = 27.9) and the group that did not receive the notifications (*M* = 31.2, *SD* = 24.0) when only active individuals were observed, but the difference was not significant (*t*(28) = 1.0, *p* = 0.319).

Comparing the proportion of tasks completed regardless of the pre-defined deadline there was almost no difference, with 78.61% of tasks accompanied with TN completed and 80.45% of tasks accompanied with NTN completed. Similarly, the completion percentage difference was not observed for tasks completed regardless of the pre-defined deadline, when we grouped the data according to whether a notification was sent out (80.1% completion) or not (76.5% completion), leading us to a conclusion that H4 and H5 should be rejected.

## 5. Discussion

Our research design carried an assumption that there is a uniform influence of tailored messages over users, depending on their dominant personality dimension. However, what we discovered points to a more nuanced story, one where within-subject results should inform and guide between-subject research, which we took into account in H6.

Users were first analyzed as a single group, but no significant differences were observed. The users, on average, completed the largest proportion of tasks with the pre-defined deadline and when receiving a TN (followed by NTN and no notification). This suggests that motivating messages in the form of push notification bring faster realization of tasks when users want to achieve the tasks in a shorter time frame.

In general, the messages had a similar average score, however, customized messages may work well for some individuals while working poorly for others. When only active users were considered—with 40 notifications or more—we observed bigger differences between TN and NTN. Afterwards, we decided to check the differences for each user individually. In the analysis of the scores of the messages we found seven interesting users who can roughly be divided into three groups, as seen in Table 2. This produces a small sample, so it should only be taken as a possible insight for future work. Looking at these groups, females in general rated the messages as more motivating than males.

Users U1 and U2 formed a group in which there was a statistically significant difference between the average scores of TN and NTN, with NTN having higher score. Excluding U2, who is without a strongly expressed personality dimension and has higher scores for NTN than TN, we observed that users among whom there was a greater difference between TN and NTN (TN motivate less than NTN) are those who had high agreeableness and lower neuroticism.

In two users, extraversion was more expressed. Such users generally do not desire using PTs [22], which could be the reason why TN did not motivate them more than NTN. Users U6 and U7 had characteristic differences in scores of TN and NTN in that they were more motivated by TN. Both users had high neuroticism and low extraversion.

From our results, we can discern two general insights: (1) Using personality-tailored messaging in a dynamic mobile domain as opposed to a static (e.g., classic marketing) domain leads to different outcomes, and it seems that there is no one-to-one mapping between domains; (2) A major reason for most of our hypotheses being false may be that messages that are deemed as persuasive on their own are not what persuades people to perform an action—e.g., even if a user gives a high score to a message for its perceived persuasiveness that does not mean that the user was persuaded by it to take an action [17]. This seems to present an important barrier for applicability of research on tailored messaging as we would have to construct a different metric for finding out what messages are persuasive—they have to be persuasive in terms of people taking actions because of them, not in terms of people finding them persuasive on their own.

To the extent of our knowledge there is no completely comparable research to ours. However, we can offer a short discussion of our findings in comparison to somewhat related research. Anagnostopoulou et al. [30] investigated the use of tailored messaging to nudge people for sustainable behavior. They used the Big Five personality traits model to obtain personality profiles of their users and personalize the mobile messages. The evaluation seemed positive, as qualitative data showed positive response to the messages, but the research was without a control group, which means that the authors could not test whether personality-tailored messages had more success than non-tailored messages. However, their evaluation seemed to go in the direction of specific (groups of) people being more influenced by personalization, which is in line with our findings. Similar to our results, Ref. [21] did not find significant effects robustly linking any of the Big Five personality traits to differences in privacy behavior caused by tailored nudges. However, they conclude that “tailored privacy nudges are likely not feasible for many small-scale applications of nudges” [21], p. 186, pointing to the fact that larger groups of people are needed to individualize nudges more specifically. In his PhD dissertation, Kaiser [31] shows that “nudges have a median effect size of 21% and that only 63% of all nudging treatments produce significant results” [31], p. x, further suggesting that only a certain group of people within the groups constructed through personality profiling are susceptible to nudges. This affirms our insight that personalization is more nuanced, i.e., that personalization works only for some personalities. Other studies suggest something similar [32].

## 6. Conclusions and Future Work

In this paper, we explored the role of personalized messages in a mobile dBCI app. We aimed to confirm that messages, personalized to personality traits, motivate users more than non-tailored ones to achieve a desired behavior. A positive finding would indicate that perceived persuasiveness from personality-tailored messaging in static domains holds true when persuading people to take action in a mobile domain. We conducted a study in which 27 volunteers used an app we created. The application’s personality-tailored motivational messages were the core tool with which we aimed to elicit persuasiveness. We recorded the information about the completion of tasks that users defined in the app and sampled the perceived motivation of messages delivered to users with the help of the experience sampling method. We found that tailoring messages only on the dominant Big Five personality trait of a user does not significantly change the outcome of their action when compared to a non-tailored message, therefore rejecting hypotheses 1–5. Following hypothesis 6, however, we observed more nuanced differences between groups of users (as discussed in Section 5).

Apart from the specific results, we believe that a major contributions of our work is also a novel dataset, which is publicly available for further scrutiny (https://gitlab.fri.uni-lj.si/lrk/personality-tailored-notifications (accessed on 20 February 2022)) as well as the notion that cross-domain transfer of personality-tailored messaging has to be explored in more nuance as transferability does not seem to be straightforward. We also want to stress the possibility that techniques that were proven to be perceived as persuasive does not necessarily mean that they will persuade people to perform an action. This last insight represents the biggest obstacle to overcome when trying to apply research in this area and produce practical outcomes.

Due to the limitations present in conducting this user study, the sample of users in whom statistically significant differences were observed is small, which stems from the entire sample size of 27, which may be too small for generalization—even though this is not an uncommon sample size in similar in situ research [30,33], while the sample size of at least 30 satisfies the Central Limit Theorem in psychology [34]. Nevertheless, the differences indicated connections, which gave us confidence that with a larger sample of users and other methods of analysis used, such as hierarchical models, they would be even more visible and validated.

In our future work, we want to adapt the application based on user feedback, collected and analyzed data and by taking current limitations into account. If conditions allow, we also want to collect larger samples of users and use more advanced methods of analysis (e.g., hierarchical models). Lastly, we would like to identify characteristic groups susceptible to personalised persuasion based on personality, as well as those groups that are not susceptible to personalised persuasion. Another direction to achieve better results is looking into user engagementresearch shows that considering user engagement promotion leads to better outcomes [35].

## Figures and Tables

**Table 1:** Table with listed hypotheses and outcome (1—Confirmed; 0—Rejected) from our analysis.

Hypothesis	Outcome
H1: The average scores from the app’s evening questionnaire for TN and NTN differ within different groups of users (e.g., different dominant personality dimensions).	0
H2: The ratios of finished tasks with pre-defined deadlines differ depending on whether the tasks are accompanied by TN or NTN.	0
H3: The ratios of finished tasks with pre-defined deadlines differ depending on whether the tasks are accompanied by notifications (either TN or NTN) or not.	0
H4: The ratios of finished tasks without considering pre-defined deadlines differ depending on whether the tasks are accompanied by TN or NTN.	0
H5: The ratios of finished tasks without considering pre-defined deadlines differ depending on whether the tasks are accompanied by notifications (either TN or NTN) or not.	0
H6: The average scores of TN and NTN differ within individuals.	1

**Table 2:** Comparison of users according to the ratings of notifications with the evening questionnaire. Levene’s test was used to infer whether the variances between the ratings of TN and NTN are equal, if so, we proceeded with *t*-test otherwise with Welch’s *t*-test (denoted by ’*’).

	*M1*	*SD1*	*M2*	*SD2*	*t*(df) = tstat	*p*	gndr	C	A	N	O	E
User 1	2.4	1.2	3.3	1.3	*t*(60) = −2.7	0.010	M	13	95	3	95	88
User 2 *	1.2	0.6	2.0	1.4	*t*(78) = −2.3	0.028	M	30	35	29	27	1
User 3	4.6	0.2	4.7	0.3	*t*(32) = −2.0	0.052	F	90	78	19	36	56
User 4	4.8	0.3	4.9	0.3	*t*(82) = −1.2	0.243	F	90	78	40	79	89
User 5	4.0	1.1	4.8	0.2	*t*(13) = −2.1	0.058	F	1	99	84	7	35
User 6 *	3.0	1.4	2.3	1.3	*t*(67) = 1.9	0.056	M	4	83	99	27	1
User 7	3.9	0.56	2.6	1.0	*t*(22) = 4.0	0.001	F	3	52	99	36	35

## Data Availability

The dataset collected and used for our research can be freely obtained on the following link: https://gitlab.fri.uni-lj.si/lrk/personality-tailored-notifications (accessed on 20 February 2022).
